# The Functions of Hepatitis B Virus Encoding Proteins: Viral Persistence and Liver Pathogenesis

**DOI:** 10.3389/fimmu.2021.691766

**Published:** 2021-08-12

**Authors:** Fenglin Zhao, Xiaoyu Xie, Xu Tan, Hongli Yu, Miaomiao Tian, Huanran Lv, Chengyong Qin, Jianni Qi, Qiang Zhu

**Affiliations:** ^1^Shandong Provincial Hospital, Cheeloo College of Medicine, Shandong University, Jinan, China; ^2^Shandong Provincial Engineering and Technological Research Center for Liver Diseases Prevention and Control, Jinan, China; ^3^Shandong Provincial Hospital Affiliated to Shandong First Medical University, Jinan, China; ^4^The First Affiliated Hospital of Xinjiang Medical University, Urumqi, China

**Keywords:** hepatitis B virus (HBV), immune response, viral escape, hepatocellular carcinoma (HCC), encoding proteins

## Abstract

About 250 million people worldwide are chronically infected with Hepatitis B virus (HBV), contributing to a large burden on public health. Despite the existence of vaccines and antiviral drugs to prevent infection and suppress viral replication respectively, chronic hepatitis B (CHB) cure remains a remote treatment goal. The viral persistence caused by HBV is account for the chronic infection which increases the risk for developing liver cirrhosis and hepatocellular carcinoma (HCC). HBV virion utilizes various strategies to escape surveillance of host immune system therefore enhancing its replication, while the precise mechanisms involved remain elusive. Accumulating evidence suggests that the proteins encoded by HBV (hepatitis B surface antigen, hepatitis B core antigen, hepatitis B envelope antigen, HBx and polymerase) play an important role in viral persistence and liver pathogenesis. This review summarizes the major findings in functions of HBV encoding proteins, illustrating how these proteins affect hepatocytes and the immune system, which may open new venues for CHB therapies.

## Introduction

Despite the widespread use of preventive vaccines has reduced new infections considerably, hepatitis B virus (HBV) infection remains a global health issue. WHO estimated that 257 million persons were chronically infected with HBV worldwide in 2015 (1). In contrast to the acute HBV infection which usually causes self-limiting and transient hepatitis, the persistent HBV infection can lead to a wide span of liver disease, like chronic hepatitis of different grades, which can progress to liver fibrosis, cirrhosis and culminate in decompensated liver disease and/or hepatocellular carcinoma (HCC) (2). According to the 2017 Global Burden of Disease Study, cirrhosis and liver cancer due to hepatitis B accounted for approximately 709400 deaths annually (3). Although diverse therapeutic drugs, such as nucleoside or nucleotide analogues (NUCs) and interferon (IFN), have been used to suppress viral replication and slow disease progression, HBV cure is still an unfeasible goal. One main reason for the antiviral treatment failure is that currently HBV covalently closed circular DNA (cccDNA) is unable to be eradicated or inactivated. CccDNA is a stable episomal template for HBV replication and transcription, its activation is facilitated by the regulatory protein HBx. The inability to eliminate cccDNA is a vital cause for HBV persistence and the subsequently liver pathogenesis (4). The other reason for treatment failure is the immune suppression induced by HBV which allows viral replication and transcription (5). And accumulating data have revealed that the HBV immunosuppressive function is at least partially mediated by the virus encoding proteins.

Several virus encoding proteins have been implicated as oncogenic proteins, especially hepatitis B envelope antigen (HBeAg) and HBx (6, 7). Among them, HBx is considered as the major carcinogenesis-related protein in HBV infection, it is indispensable to initiate and maintain virus replication. Meanwhile HBx also has promiscuous functions on diverse cellular events, such as epigenetic modifications, ubiquitination, autophagy and non-coding RNA (ncRNA) regulation, which ultimately contribute to viral persistence, hepatic inflammation, fibrosis and hepatocarcinogenesis.

However, the mechanisms of HBV infection chronicity are still elusive and might be multifactorial. The HBV encoding proteins may play a crucial part in the chronicity of HBV infection. In this review, we summarize the recent evidence on HBV encoding proteins, with a focus on their functions in viral persistence and liver pathogenesis. It aims to deepen the comprehension on the underlying mechanisms of HBV encoding proteins induced viral persistence and liver pathogenesis therefore provide reasonable strategies for CHB therapies.

## HBV Life Cycle

The hepatitis B virion is a small enveloped DNA virus belonging to the Hepadnaviridae family, it infects and replicates mainly in hepatocytes (8). HBV virion has an outer lipoprotein envelope formed by hepatitis B surface antigen (HBsAg) and an icosahedral nucleocapsid formed by hepatitis B core antigen (HBcAg) which encloses a partially double-stranded, relaxed circular DNA (rcDNA) genome of 3.2kb. The virus genome is organized in a highly condensed manner, all the genes are encoded within four partially overlapping open reading frames (ORFs), termed S (surface), C (core), P (polymerase) and X (X protein) and include 7 proteins: HBcAg, HBeAg, HBx, polymerase (pol) and the three different sizes HBsAg- the small (S), medium (M) and large (L) proteins (8, 9).

The virus binds to heparansulfate proteoglycans (HSPGs) to initiate infection, this is a low-affinity binding reaction which is reversible (10). Subsequently, HBV interacts with the sodium taurocholate co-transporting polypeptide (NTCP) receptors on the surface of hepatocytes *via* the PreS1 domain of large HBsAg protein with high affinity to trigger viral internalization (11). And epidermal growth factor receptor (EGFR), a co-receptor of NTCP, is also required for viral internalization (12, 13). After being endocytosed, HBV viron releases its DNA-containing nucleocapsid into the cytoplasm. After nucleocapsid disassembly, HBV DNA is subsequently transported to the nucleus by crossing of the nuclear pore complex in an importin-α/-β dependent manner (14). Then rcDNA is remodeled into a cccDNA minichromosome in a host cell DNA repair response dependent way (15). In the meantime, HBV DNA can integrate into the host genome, which does not necessarily take place in all hepatocytes. However, the specific molecular mechanisms of nucleocapsid disassembly and cccDNA biosynthesis are still elusive. Nuclear cccDNA molecules associate with cellular histone and non-histone proteins forming a nucleosomally organized minichromosome (16, 17). The cccDNA minichromosome persists in the nucleus as a central transcription template, utilizing host RNA polymerase II machinery to generate all HBV transcripts, including the 3.5kb pre-genomic RNA (pgRNA) which encodes viral polymerase and HBcAg, the 3.5kb preCore mRNA encoding HBeAg, the 2.4kb preS1 and 2.1kb preS2/S mRNA encoding the three different sizes HBsAg, and a 0.7kb mRNA encoding the regulatory protein HBx (9).

**Figure 1:**
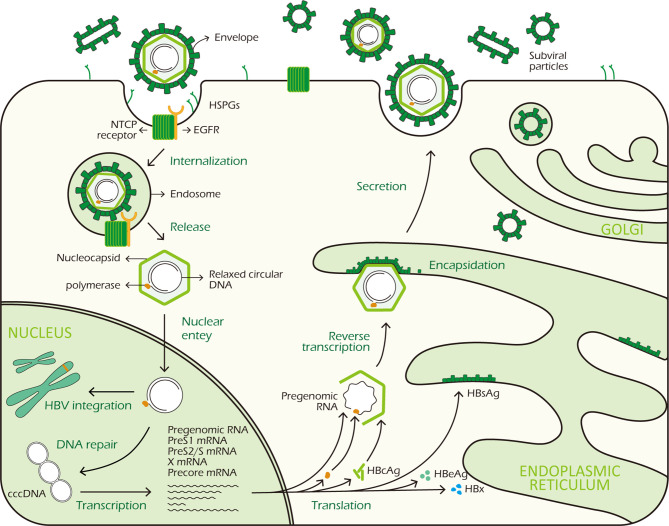
HBV life cycle. HBV virus binds to HSPGs with low affinity to initiate infection, subsequently it interacts with NTCP receptor on the surface of hepatocyte to trigger viral internalization in a EGFR dependent manner. After being endocytosed, the virus releases its DNA-containing nucleocapsid into the hepatocyte cytoplasm which is subsequently transported to the nucleus. In the nucleus, the rcDNA is repaired and converted to cccDNA. At the same time, integration of viral DNA into host genome also takes place. The cccDNA minichromosome persists in the nucleus and functions as a transcription template for viral RNA transcription. It codes 7 viral proteins required for replication: 3 different sizes HBsAg, HBcAg, HBeAg, HBx and polymerase. In the cytoplasm, HBcAg proteins self-assemble into icosahedral nucleocapsid to package viral polymerase and pgRNA. After reverse transcription in nucleocapsid, HBV genome-containing capsid binds to HBsAg proteins in the ER for encapsidation. Finally, mature HBV viruses exit the hepatocytes *via* MVBs.

In the cytoplasm, HBcAg proteins package viral polymerase and pgRNA to assemble into nucleocapsid. The pgRNA is reverse transcribed by the viral DNA polymerase to produce the rcDNA (8). To egress from cells, surface proteins are required for encapsidation, excluding M-HBsAg which is not necessary for virion morphogenesis and secretion. HBV genome-containing nucleocapsid binds to HBsAg proteins in the endoplasmic reticulum (ER) before exiting the hepatocytes through *via* multivesicular bodies (MVBs) (18). **Figure 1** shows the HBV life cycle.

## HBsAg

The viral envelope is formed by HBsAg. The three different sizes HBsAg are translated from two HBV subgenomic mRNA transcripts, L-HBsAg from the preS1 mRNA and M-HBsAg and S-HBsAg from the preS2/S mRNA, in the ER (9). Excluding the envelope, HBsAg proteins could assemble to non-infectious spherical and filamentous subviral particles (SVPs), approximately 22 nm in diameter. These non-infectious SVPs are secreted in excess, approximately 1,000- to 100,000-fold higher than the infectious particles (19).

HBsAg is a multifunctional protein. To date, it is reported that HBsAg could activate unfolded protein response which might sensitize hepatocytes to cell death and lead to possible subsequent cellular premalignant changes, indicating HBsAg might related to hepatic inflammation and hepatocellular carcinogenesis (20). And multiple data indicate that HBsAg can cause the deregulation of both innate and adaptive immune systems *via* interacting with immune cells and non-immune cells, contributing to not only the ability of HBV to escape and control the host immune system for its persistence, but also the liver damage during the chronic phase.

### The Effect of HBsAg on Innate Immune

Innate immunity is viewed as the first line of defense against various pathogens, including viruses, and it also interacts closely with adaptive immune system. At the early stage of innate immune response, pathogen recognition receptors (PRRs) on immune cells non-specifically detect and recognize pathogen-associated molecular patterns (PAMP) of pathogens, leading to activation of various signaling pathways for eliminating pathogens (21). HBsAg is capable of compromising the normal immune responses of many innate immune cells, such as monocytes/macrophages, kupffer cells (KCs), natural kill (NK) cells, Dendritic cells (DCs) and myeloid-derived suppressor cells (MDSCs) (22–27).

Among PRRs, Toll-like receptors (TLRs) are considered critical for virus recognition and antiviral immune response. A study by Sen Wang et al. demonstrated that in M/MΦs, HBsAg selectively suppressed the TLR2 ligand (Pam3csk4)-stimulated interleukin (IL)-12 production *via* impairing the JNK–MAPK pathway in a dose-dependent manner (22). IL-12 is an immunoregulatory cytokine which could facilitate T-helper (Th) 1 cell response and T cell proliferation, therefore bridging innate and adaptive immune. Whilst c-Jun is a JNK kinase downstream transcriptional factor, it regulates IL-12 transcriptional activity. Impaired phosphorylation of c-Jun caused by HBsAg could cause a reduction of IL-12 production (28, 29). It reveals a mechanism that HBsAg evades the host innate immune response and hinders Th1 adaptive immunity by inhibiting IL-12 (22).

Meanwhile, as the resident macrophages in liver, KCs play a critical role in the innate immune response (30). KCs is able to detect HBsAg *in vivo* and *in vitro*, leading to HBsAg internalization and production of IL-6, TNF, and CXCL8 (C-X-C motif chemokine ligand 8). However, HBsAg did not induce an activated phenotype of KC based on costimulatory molecule and major histocompatibility complex (MHC) expression. And HBsAg also functionally interacts with *in vitro*–generated macrophages which could lead to NK cell activation. Generally, KCs in liver tightly adhere to sinusoidal endothelial cells and often interact with NK cells. NK cells approximately account for 30% of liver lymphocytes which can identify and remove viral infected cells without MHC restriction effectively. Thus, the events above may support HBV-specific immune responses during the early infection phase, but may also contribute to liver damage during the chronic phase (23). In addition, it has been reported that HBV can specifically decrease absent in melanoma 2 (AIM2) transcripts in KCs and differentiated monocytes. AIM2 inflammasome is able to activate KCs to produce IL-1β and IL-18, which activates IL-8 transcription and hepatic NK cell activity respectively. HBsAg can abrogate AIM2 inflammasome responses by deregulating interferon regulatory factor (IRF) 7 expression in KCs which reduces IRF7 binding to the AIM2 promoter, leading to innate immune responses escape (24).

In terms of DCs, they are specific antigen-presenting cells which play a key role in antiviral immunity by functioning as messengers between innate and adaptive immune responses. After antigen recognition, DCs process antigens, subsequently present antigens and increase costimulatory molecules expression as well as cytokine production, ultimately activate naïve T cells to stimulate adaptive immunity (31). Myeloid dendritic cell (mDC) could induce strong T cell proliferation to initiate antiviral responses. HBsAg can be internalized by mDC and inhibit the up-regulation of costimulatory molecules during mDC maturation which impairs mDC maturation and function, thereby giving rise to more tolerogenic mDC (25). Plasmacytoid dendritic cells (pDCs) are the main IFN-α producing cells among peripheral blood mononuclear cells (PBMCs), and play an important role in the antiviral immune response. HBsAg is able to induce monocytes to secrete TNF-α and IL-10 and these cytokines down-regulate the TLR9 expression in pDCs, leading to decreased production of IFN-α by pDCs (26). These immune regulatory effects of HBsAg particles on the function of DC can be considered as part of the mechanism of HBV immunity escape.

Besides, MDSCs also can be modulated by HBsAg (27). MDSCs are derived from myeloid progenitor cells, they are capable of inhibiting T lymphocyte activation thus functioning as negative regulators of immune responses (32). HBsAg promotes the ERK/IL-6/STAT3 (signal transducer and activator of transcription 3)-dependent differentiation of MDSCs from monocytes, leading to T cell function impairment (27).

### The Effect of HBsAg on Adaptive Immune

Adaptive immunity is a sophisticated part of the immune system. In response to the altering challenges of various pathogens, adaptive immunity can undergo pathogen-specific changes to fight the infection, which are different from the pathogen-unspecific responses of innate immunity. During HBV infection, the outcome of infection is ultimately determined by HBV-specific antibody producing B cells and functional HBV-specific T cells in adaptive immunity (33). A defective adaptive immune response is responsible for HBV control failure and establishment of chronic infection. Yet the adaptive immunity, especially T and B cells function, is harassed by HBsAg in HBV infection.

**Figure 2:**
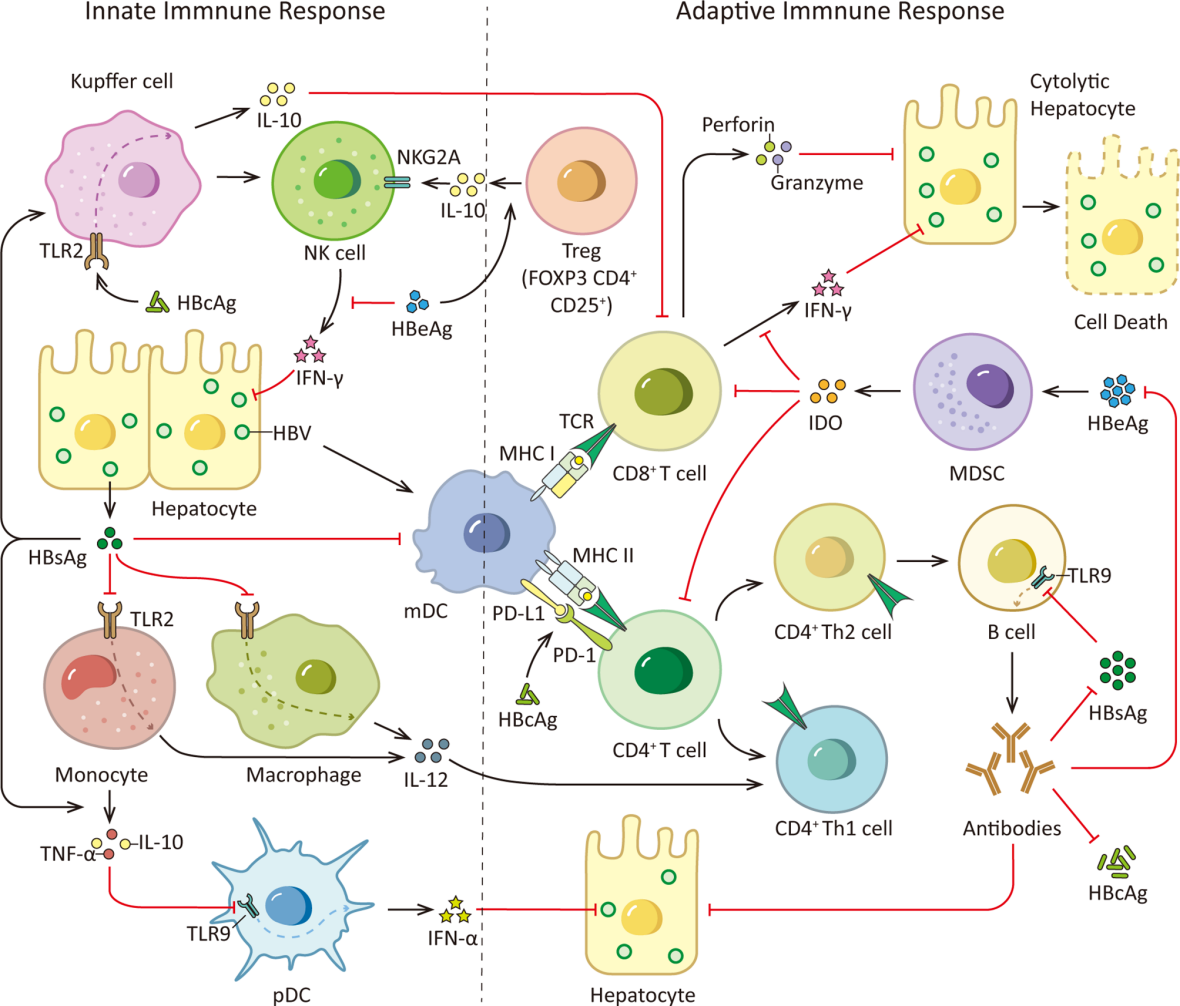
HBsAg, HBcAg, HBeAg and immune responses. During HBV infection, the viral PAMPs can be detected and recognized by innate immune cells, such as Kupffer cells, macrophages, monocytes and pDCs, contributing to the release of cytokines and the subsequent activation of other immune cells. However, HBV virus is able to counter this initial immune response with its proteome, especially HBsAg, HBeAg and HBcAg. HBsAg can selectively suppress the TLR2 ligand-stimulated IL-12 production in monocytes and macrophages, thus hinder IL-12 induced Th1 cell response and T cell proliferation. And it is able to induce monocytes to secrete TNF-α and IL-10 and down-regulate the TLR9 expression on pDCs, leading to decreased production of IFN-α by pDCs. Moreover, HBsAg inhibits up-regulation of costimulatory molecules during mDC maturation thus hinders mDC maturation and function. In addition, HBcAg can elevate IL-10 production *via* stimulating TLR2 on KCs which subsequently induce anti-HBV CD8+ T cell exhaustion. Viral clearance requires coordinated response of both the innate and adaptive immune system. Among adaptive immunity, vigorous T and B cell responses to the HBV encoding proteins is indispensable. While viral persistence is associated with impaired HBV-specific adaptive immune response. HBsAg decreases TLR9 transcription in B cells, resulting in diminished B cells proliferation, differentiation and antibodies production. Besides, HBcAg induces hyper-expression of PD-1 on CD4^+^ T cells and up-regulates the PD-L1 expression on monocyte-derived DC, leading to a coinhibitory signal to T cells. What’s more, HBeAg is able to induce elevated IL-10 production in Tregs, increasing expression of inhibitory receptor NKG2A on NK cells. And HBeAg induces mMDSCs expansion and up-regulates immune suppressor molecules IDO in mMDSCs to impair the proliferation of CD4+ and CD8+ T cells and IFN-γ production. TCR, T cell receptor.

A recent study demonstrated that in patients with chronic HBV infection, there is a correlation between the age of the patients and HBs-specific T cells. They found HBs-specific T cells declined with patients’ age which corresponded to the duration of HBsAg exposure rather than the quantity of HBsAg, and the global T cell profile was not affected (34). And it has been reported that HBsAg is able to attenuate TLR3-mediated antiviral effects by suppressing IRF-3, NF-κB and mitogen-activated protein kinases (MAPKs) in nonparenchymal liver cells to inhibit T lymphocytes activation in mice (35). Moreover, antibody responses could be induced by different HBV proteins. Especially, anti-HBs antibodies are thought to be protective. By neutralizing HBsAg, anti-HBs could prevent NTCP induced HBV entry to hepatocyte. However, SVPs may exert functions as decoys for the host immune system by neutralizing anti-HBs antibodies, thereby promoting virus spread and persistence in the infected host (36, 37). Furthermore, recent study demonstrates that HBsAg has the ability to dampen B cells function. HBsAg decreases TLR9 transcription in B cells *via* preventing cAMP-responsive element-binding protein (CREB) phosphorylation and binding to cAMP response element (CRE), subsequently inhibiting TLR9 promoter activity. Innate sensor TLR9 activation is crucial to B cell activity. In response to TLR9 stimulation, B cells proliferate, differentiate, produce antibodies and secrete cytokines, such as IL-2, IL-4, TNF-α, and IL-6. Internalized HBsAg in human B cells decreases protein level of protein kinase A (PKA) which is critical for CREB phosphorylation at Ser, thus prevents TLR9 transcription, providing a novel insights into HBV immune escape strategies (38). The immune responses triggered by HBsAg are illustrated in **Figure 2**.

## HBcAg

HBcAg is a 183 residue protein, helical homodimer which performs multiple functions on the viral lifecycle. In hepatocyte cytoplasm, HBcAg can self-assemble into the 120-dimer icosahedral viral nucleocapsid to enclose the HBV genome and DNA polymerase (39). Besides its structural role, its regulatory functions in HBV life cycle and host immune system have been better understood in recent years.

There are recent advances in understanding the interaction between HBcAg and host proteins and the regulation role of HBcAg in the transcription of viral genes. In nucleus, HBcAg can bind to nuclear HBV DNA and regulate its epigenetics (39, 40). Chong and colleagues showed that HBcAg interacts with cccDNA and associated histone acetyltransferases (HATs) through its C-terminal domain (CTD) arginine-rich clusters, therefore regulates HBV transcription (41). And it also has been reported that HBcAg could increase HBV Enhancer (Enh) I transcriptional activation *via* the CRE/CREB/CBP(CREB binding protein) pathway in the HepG2 cell line (42). As a member of APOBEC family, cytidine deaminase APOBEC3A could deaminate and degradate foreign nuclear DNA, including HBV cccDNA. Julie Lucifora and colleagues have demonstrated that HBcAg is able to interact with APOBEC3A and facilitate the binding between APOBEC3A and cccDNA which leads to cccDNA degradation (43).

Moreover, as a stimulus possessing strong immunogenicity, HBcAg is able to induce antigen-specific T-cell responses which are critical for HBV infection control. However, HBcAg also has evolved to cause HBV immune escape by utilizing multifaceted strategies. For example, HBcAg could induce a strong Th17 cell response which is important in T-cell immunity against HBV infection. HBcAg promotes IL-17A, IL-22, IL-23, IL-6, transforming growth factor (TGF)-β and IL-10 production by PBMCs. While immunosuppressive cytokine IL-10 negatively regulates the development of Th17 cells, which contributes to the persistence and progression of HBV infection (44).

It is well-known that programmed cell death-1 (PD-1) is a member of the CD28 family, it generally functions as an inhibitory costimulation molecule in immunity modulation and its hyper-expression is viewed as a hallmark of exhausted T cells. A study demonstrated that compared with healthy individuals, the PD-1 expression level on CD4^+^T cells in peripheral blood was significantly higher in CHB patients. This hyper-expression of PD-1 on CD4^+^T cells was induced by HBcAg *via* JNK, ERK and phosphoinositide 3-kinase (PI3K)/AKT signaling pathways, resulting in impaired adaptive immunity (45). While B7-H1 (also called programmed death ligand 1, PD-L1) also plays an important role due to its ability to bind to PD-1 and thereby deliver a coinhibitory signal to T cells. In CHB patients, the frequencies of peripheral DCs and mDCs were reduced and B7-H1 expression on DCs and mDCs were up-regulated (46, 47). And it was shown *in vitro* experiments that the apoptosis of monocyte-derived DCs (Mo-DC, cells express the same markers as mDC) and B7-H1 expression on Mo-DCs were induced by HBcAg, which could be the underlying mechanism for the observed effects in CHB patients (47).

Besides, HBcAg could elevate IL-10 production *via* stimulating TLR2 on KCs in mice which subsequently induced anti-HBV CD8+ T cell exhaustion in HBV-carrier mice, leading to immune tolerance in liver (48). The immune responses triggered by HBcAg are illustrated in **Figure 2**. However, in our previous research, we found HBcAg could not effectively induce macrophage activation and participated in the liver damage caused by it (49–51). Therefore, HBcAg is a multifunctional protein, it is not only involved in viral nucleocapsid formation, but also associated with cccDNA epigenetic modulation and HBV immune evasion. Further research on HBcAg is needed for the better understanding and novel implications for the development of HBcAg-targeted antiviral drugs.

## HBeAg

HBeAg is translated from the preCore mRNA and it is processed in ER (52). It is regarded as an HBV accessory protein which is not necessary for viral replication or infection whereas it is considered as a marker of active viral replication and associates with the establishment of chronic infection, hepatic inflammatory injury and HCC development. According to a study by Xin Yu, lipopolysaccharide (LPS) induced NLRP3 inflammasome activation and IL-1β production in KCs was inhibited by HBeAg *via* suppressing NF-κB pathway and ROS production (53). HBeAg suppresses both NF-κB and IFN-β activation by specifically interacting and co-localizing with Toll/IL-1 receptor (TIR) proteins. Importantly, it appears HBeAg disrupts TIR : TIR homotypic dimerization to affect the downstream TLR-mediated signaling, which is a possible mechanism for the viral persistence and immune tolerance (54).

In addition, NK cells are enriched in liver, and they are potential to be a major source of IFN-γ. Reduced IFN-γ production due to NK cells dysfunction could impair the noncytolytic antiviral capacity. As a recent study demonstrates that HBeAg is able to increase expression of inhibitory receptor NKG2A on NK cells by inducing IL-10 production in regulatory T cells (Tregs), which leads to NK cells dysfunction during CHB infection (55). Besides, it is shown *in vitro* that HBeAg protein inhibits IL-18-mediated NF-κB signaling to down-regulate IFN-γ production in NK cells with a dose-dependent mechanism (56). It has been reported that in CHB patients, Major vault protein (MVP) expression level in both hepatocytes and immune cells is up-regulated. MVP is a MyD88-associated protein which could be induced by viruses, its interaction with MyD88 positively regulates MyD88-induced activation of NF-κB and IFN-β. Both HBeAg and HBsAg could directly bind to MVP thus hinder the interaction between MyD88 and MVP to limit downstream IFN signaling. It reveals a possible mechanism for HBV evading immune responses *via* disrupting the antiviral activity of IFN (57). What’s more, HBeAg could induce monocytic MDSCs (mMDSCs) expansion and up-regulate immune suppressor molecules indoleamine-2, 3-dioxygenase (IDO) in mMDSCs to impair the proliferation of CD4+ and CD8+ T cells and IFN-γ production in CHB patients (58). These studies provide novel sights for HBV evading immune responses which may contribute to the establishment of viral persistence and immune tolerance (see **Figure 2**).

Of note, HBeAg is also capable of drawing advantages from immature immune system of children to facilitate the HBV persistence. In contrast to horizontal transmission of HBV between adults which generally leads to self-limited acute infection, the vertical transmission from mother to child frequently leads to viral persistence. By developing a mouse model, Tian et al. found that maternal HBeAg could condition Kupffer cells of the offspring to undergo M2 polarization and express an increased level of PD-L1, leading to the M2-like anti-inflammatory response of KCs and suppressed CD8+ cytotoxic T lymphocyte (CTL) response, which resulted in immune suppression in offspring (59). Nevertheless, our previous study has verified that HBeAg, but not HBcAg and HBsAg, played a pivotal role in macrophage activation and induced multiple production of inflammatory factors to accelerate liver injury (49, 50). The impact of HBeAg on the immature immune system needs further research which may contribute to better understanding of the chronicity of HBV infection.

Furthermore, beyond the immune regulatory function, HBeAg has been considered playing a critical role in tumor growth and hepatocarcinogenesis in recent years (60). HBeAg and its precursor preCore/p22 interact with NUMB to promote E3 ubiquitin ligase HDM2-mediated degradation of p53 and inhibit the translocation of p53 from cytosol to nucleus, which contributes to HCC development (7). Thus, the research in recent years sheds light on that HBeAg is more than a viral nonstructural protein, it plays an important role in the establishment of immune tolerance, liver injury and hepatocellular carcinogenesis.

## HBV DNA Polymerase

The essential function of HBV DNA polymerase is initiating viral replication by reverse transcription. First the polymerase synthesizes the single-stranded linear DNA by using the pgRNA as a template, subsequently synthesizes partially double-stranded rcDNA (37). Recently, growing evidence has indicated that HBV polymerase exerts other functions by various mechanisms (61–63).

It has been reported that HBV polymerase acts as an immune regulatory molecule that counteracts host innate immune response, contributing to innate immune escape (61). After viral infection, PRR signaling could be activated, leading to IRF activation and IFN production. HBV polymerase could bind to DDX3 DEAD box RNA helicase to disrupt IκB kinase-ϵ (IKKϵ)-DDX3 interaction, thus inhibit TANK-binding kinase 1 (TBK1)/IKKϵ activity and consequently the phosphorylation and activation of IRF3/7, leading to inhibition of IFN-β production in human hepatocytes (61). Moreover, a study by Chen et al. demonstrated that HBV polymerase impaired IFN-α–induced STAT activation through inhibiting importin-α5 and protein kinase C-δ. This study provides a better understanding of how DNA polymerase functions in HBV-mediated resistance of IFN-α treatment (62). Furthermore, a recent study has demonstrated that HBV polymerase attenuates HBV replication *via* activating the CREB1-HOTTIP-HOXA13 axis. The polymerase enhances CREB1 expression, which promotes long non-coding RNA (lncRNA) HOTTIP expression. And HOXA13, which is significantly up-regulated by HOTTIP, binds directly to Enh I/Xp to inhibit generation of HBsAg and HBeAg and HBV genome replication. This process may attenuate host cell injury to contribute to the development of chronic HBV infection and the pathogenesis of HBV-related diseases (63).

## HBx

The viral regulatory protein HBx is a 154 amino acid long protein (64). It is localized in diverse subcellular compartments, ranging from cytoplasm to nucleus, and mitochondria. And HBx in different localization may confer different functions (64, 65). HBx has various functions on multiple cellular events, including transactivator of the viral and cellular promoters, epigenetic modifications, ubiquitination, autophagy and non-coding RNA regulation, to promote hepatocellular carcinogenesis, stabilization and activation of cccDNA (64, 66). Growing evidence implicates that HBx plays an important role in viral persistence and liver pathogenesis, especially in hepatocarcinogenesis.

### HBx and Transcriptional Activation

Multiple data indicate that the regulatory protein HBx transactivates many viral and host promoters in a direct or indirect way which is generally associated with the deregulation of signal-transduction pathways. And such property of HBx raises the possibility of the development of HBV-related HCC (67–72).

Previous study has shown that HBx is able to bind to the promoter region of Yes-associated protein (YAP) by interacting with CREB, leading to up-regulation of YAP which is a key driver oncogene in the promotion of hepatoma cells growth and a downstream effector of the Hippo pathway. Ultimately, HBx-mediated up-regulation of YAP could lead to hepatoma cells growth in the development of HCC (67). Other research has reported that in hepatoma cells, HBx increases IL-34 expression in a transcription factor CCAAT/enhancer-binding protein α (CEBP/α)-dependent way. HBx is able to up-regulate CEBP/α by activating PI3-K and NF-κB pathways to facilitate IL-34 expression. Furthermore, IL-34 elevated by HBx enhances the proliferation and migration of HCC cells *via* colony-stimulating factor 1 receptor (CSF1-R) and CD138, and IL-34 contributes to the activation of ERK and STAT3 pathways as well as the up-regulation of Bcl-xl and c-Myc mediated by HBx (68). In addition, HBx up-regulated Forkhead box M1 (FoxM1) expression through ERK/CREB signaling pathway, contributing to invasion and metastasis of hepatoma cells. FoxM1 could transactivate matrix metalloproteinase (MMP) 7, RhoC, and Rho-kinase 1 (ROCK1) expression, leading to the progression of HBV-associated HCC (69). Moreover, a recent study showed that HBx interacted with the Src substrate cortactin (CTTN) in the cytoplasm, resulting in the up-regulation of CREB1. In MHCC-LM3 and HepG2 cells, CREB1 overexpression significantly reduced E-cadherin expression and increased vimentin and MMP9 levels which promotes the proliferation and migration of HCC cells (70).

Additionally, HBx enhances androgen receptor (AR) activity in an androgen-dependent manner. HBx can facilitate AR dimerization and increase its transactivation ability by activating c-Src protein kinases and inhibiting GSK3β. And there are two conserved androgen response elements (ARE) within the Enh I of HBV, suggesting an AR-mediated stimulation of overall HBV transcription, by which viral replication is promoted and protein production, including HBx, is elevated. Therefore, a positive feedback loop between HBx and AR is established in HBV-infected male hepatocytes, which may play an important role in hepatocarcinogenesis (71). Furthermore, a study performed by Xu et al. demonstrated that HBx conferred resistance of hepatoma cells to anoikis. Anoikis is a specialized form of apoptosis that occurs on cells due to inadequate or inappropriate cell-matrix interactions (73). HBx up-regulates and hyperactivates the serine/threonine p21 activated kinase (PAK) 1. PAK1 regulates cytoskeletal dynamics and protects cells from anoikis. Mitochondrial-localized PAK1 interacts with Bcl2 to allow hepatoma cells to become resistant to anoikis which might promote progression of HCC in patients with chronic HBV infection (72). Therefore, HBx can act as an oncoprotein by exerting functions as a promiscuous transactivator of both viral and host promoters.

### HBx and Epigenetic Modulation

Soon after HBV virion infecting a cell, the rcDNA is converted to cccDNA. The cccDNA minichromosome exists in the nucleus as an episomal DNA template, binding with histone and non-histone proteins. The inability to eradicate or inactivate cccDNA is a major reason for anti-viral treatment failure (4). Accumulating evidence indicates that epigenetic modifications occur on both cccDNA and host genome. And the regulatory protein HBx is needed for epigenetic modifications of cccDNA to initiate and maintain viral RNA transcription (74). Generally, the epigenetic modifications mediated by HBx are likely contributed to viral persistence and/or carcinogenesis.

Gene expression of chromatin is partially regulated through histone modification. Compelling evidence has demonstrated that histone modifications of cccDNA can be mediated by different types of histone-modifying enzymes, such as lysine methyltransferases (KMTs), protein arginine methyltransferases (PRMTs), and HATs (histone acetyltransferases). For example, the recruitment of HATs, such as CBP, p300, and the p300/CBP-associated factor (PCAF), on cccDNA is involved in histone acetylation and active cccDNA transcription. It has been shown that HBx plays a critical role in promoting the recruitment of these HATs for epigenetically regulating cccDNA function (75).

SETDB1, a histone lysine methyltransferases, induces methylation of H3 on lysine 9 (H3K9me3) which is associated with gene silencing. Through interaction with H3K9me3, heterochromatin protein 1 factors (HP1) are recruited to cccDNA then contribute to the transcriptional repression of HBV. HBx can relieve this repression therefore establish active cccDNA minichromosomes in hepatocytes (76). Recently, Weiwu Gao and colleagues have demonstrated that the HBx-WDR5-H3K4me3 axis contributes to liver carcinogenesis (77). WD repeat domain 5 protein (WDR5) is a core subunit of histone H3 lysine 4 methyltransferase complexes. HBx inhibits damage-specific DNA-binding protein 1 (DDB1)/cullin-4 (CUL4) E3 ligase induced degradation of WDR5. HBx elevates WDR5 protein levels by competitive interaction with DDB1 and recruits WDR5 to recognize chromatin, leading to increased histone H3 lysine 4 trimethylation (H3K4me3) modification, a marker of gene activation, on host and HBV chromatin (77, 78). In addition, it is known that enhancer of zeste homolog 2 (EZH2) is a histone trimethyltransferase, it catalyses a repressive mark, the methyl groups at lysine 27 of histone H3 (H3K27me3) on cccDNA and host genes. HBx could induce a reduction of EZH2 on cccDNA and host genes by modulating lncRNA DLEU2. The HBx-DLEU2 interaction could replace EZH2 from chromatin, leading to active viral replication and host cancer-related gene transcription (79).

Besides, PRMT1 negatively regulates HBV transcription through its direct recruitment to the cccDNA, HBx could relieve inhibitory activity of PRMT1 to promote viral replication (80). However, the asymmetric dimethylation on H4R3 (H4R3me2a) catalyzed by PRMT1, has been recognized as a gene activation marker. Wen Zhang and colleagues suggested that positive correlation between the status of H4R3me2a and cccDNA transcriptional activity was not found. Therefore, it is possible that the repressive effect of PRMT1 on HBV transcription act through other target proteins rather than H4R3me2a cccDNA modification (81).

**Figure 3:**
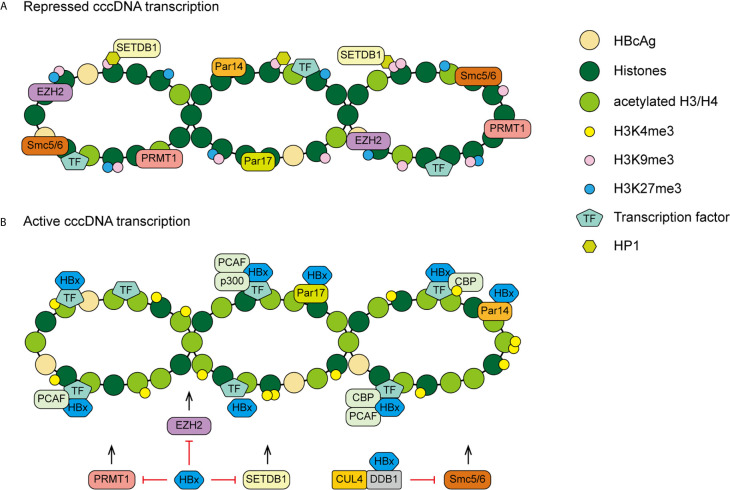
A proposed model of HBx mediated cccDNA minichromosome epigenetic modulation. **(A)** In the absence of HBx, cccDNA-bound histones are hypoacetylated and the host restriction factors are recruited on the cccDNA, contributing to repressed gene transcription and viral replication. **(B)** In the presence of HBx, HATs are recruited to cccDNA and host restriction factors, such as PRMT1, EZH2, SETDB1 and Smc5/6, are inhibited by HBx, leading to hyperacetylation of cccDNA-bound histones and active cccDNA transcription.

The mechanism of HBx acting on cccDNA is complex. Except histone modification, there are many other HBx mediated epigenetic modifications. The structural maintenance of chromosomes (Smc) complex Smc5/6 is a restriction factor selectively binding to extrachromosomal DNA. The study of Decorsiere et al. revealed that HBx targeted Smc5/6 for degradation by hijacking DDB1/CUL4 E3 ubiquitin ligase. Thus, the degradation of Smc5/6 complex induced by HBx could relieve the restriction to promote HBV gene expression (82). A recent study has demonstrated that parvulin 14 (Par14) and parvulin 17 (Par17) proteins bind to cccDNA and therefore upregulate HBV replication in an HBx-dependent way. The HBx-parvulin14/parvulin17-cccDNA interaction promotes viral replication from cccDNA to virion (83). In addition, DNA methylation often occurs within CpG dinucleotides by the activity of DNA methyltransferases (DNMTs). HBx is able to up-regulate the mRNA and protein expression levels of DNMT3A/3B in human hepatocytes, enhancing suppressors of cytokine signaling-1 (SOCS-1) CpG island methylation which leads to the activation of oncogenes (84). HBx also recruits histone deacetylase 1 (HDAC1) to the promoter of DNMT3L and DNMT3A to down-regulate their expression, inducing hypomethylation of distal intragenic CpG islands (85). **Figure 3** shows the HBx mediated epigenetic modulation on cccDNA.

### HBx and Ubiquitination

Ubiquitination is a crucial cellular regulatory mechanism for controlling physiological and pathological cellular events, which can induce target protein degradation and other cellular events. Ubiquitin covalently bonds to lysine residues on target proteins by a cascade of enzymatic reactions carried out by E1 (activation), E2 (conjugation) and E3 (ligation) enzymes, which contributes to degradation of target proteins (86, 87). Previous studies have revealed that HBx is able to regulate the process of ubiquitination *via* various strategies, which could lead to viral persistence, liver fibrosis and HCC development.

Paired box 8 (PAX8) could exert functions as an oncogenic factor for the progression of HCC. S-phase kinase-associated protein 2 (Skp2) is a component of the SCF ubiquitin ligase complex, it directly interacts with PAX8 and targets lysine 275 of PAX8 for ubiquitination and degradation. A recent study demonstrates that HBx stabilizes PAX8 by inhibiting the Skp2-dependent ubiquitination in hepatoma cells, thereby contributing to the cell clonogenicity and proliferation and its growth *in vivo*, which might promotes the progression of HBV-related HCC (88). Protein SH2 domain containing inositol 5-phosphatase 2 (SHIP2) is an enzyme which dephosphorylate the 5 position of PI(3,4,5)P3, a negative regulator of PI3K and insulin signaling. It is shown *in vitro* experiments that HBx induces Skp2 expression and thereby down-regulates SHIP2 by inducing a notable accumulation of polyubiquitinated SHIP2 in HCC cells. The down-regulation of SHIP2 leads to HCC cell migration, elevated glucose uptake and drug resistance to 5-Fluorouracil (5-FU) (89).

Moreover, Xian Lin and colleagues found that HBx could promote HCC invasion by inhibiting F-box and WD repeat domain containing 7α (Fbw7α) mediated ubiquitination and degradation of breast cancer 1 (AIB1) protein in human HCC cell lines (90). Protein AIB1 is a member of the p160 family, it can enhance a variety of transcription factors, such as activator protein-1 (AP-1) and NF-κB (91). HBx interacts with AIB1 protein to prevent the interaction between ubiquitin ligase Fbw7α and AIB1, therefore AIB1 protein level is increased (90). Moreover, both NF-κB and AP-1 act as major regulators to activate the promoter of the MMP9 gene (92). HBx and AIB1 are recruited to MMP9 promoter to up-regulate MMP9 expression cooperatively. This cross-talk between HBx and AIB1 promotes invasiveness of HCC cells (90). HBx could also interact with non-muscle myosin heavy chain IIA (MYH9) and up-regulate it *via* modulating glycogen synthase kinase 3β (GSK3β)/β-catenin/c-Jun signaling pathway, contributing to promoted tumor stemness in HCC cells. MYH9 recruits the E3 ubiquitin ligase TRAF6 to GSK3β, acting as a scaffold in the β-catenin degradation complex, thus facilitating GSK3β degradation which promotes the activity of Wnt/β-catenin signaling (93). Furthermore, HBx inhibits the self-ubiquitination of E2-EPF ubiquitin carrier protein (UCP), but promotes UCP-mediated von Hippel–Lindau protein (pVHL) ubiquitination by forming a ternary complex with UCP and pVHL. The pVHL is a tumor suppressor protein, it promotes the degradation of hypoxia-inducible factor (HIF)-1α and -2α by serving as the substrate recognition module of the E3 ubiquitin ligase complex. Thus, the decreased pVHL level leads to stabilization of HIF-1α and -2α, thereby facilitating cell proliferation and invasion in HCC cells and mice (94). And HIF-1α stabilized by HBx is able to activate the transcription of lysyl oxidase (LOX) family which deaminates lysines of the collagen in the extracellular matrix (ECM) resulting in crosslinking of collagen. The most direct pathological consequence of collagen crosslinking is liver fibrosis and cirrhosis. Besides, the remodeled collagen can promote cancer cells invasion (95). Moreover, HBx up-regulates E3 ubiquitin ligase Male-specific lethal 2 (MSL2) by activating YAP/FoxA1 signaling in hepatoma cells. APOBEC3B is a cytidine deaminase which could the deaminate and partial degrade HBV cccDNA without affecting the host genome (43, 96). MSL2 could maintain HBV cccDNA stability *via* ubiquitination and degradation of APOBEC3B therefore built a cccDNA persistence reservoir. And the elevated cccDNA stability contributes to raised HBV virus protein level, including HBx. Ultimately, A positive feedback loop of HBx/MSL2/cccDNA/HBV is formed, which might promote viral persistence and hepatocarcinogenesis (96). And as mentioned above, HBx could also regulate epigenetic modifications *via* the ubiquitination degradation system. HBx could increase H3K4me3 modification on host and HBV chromatin and promote the degradation of Smc5/6 complex by hijacking DDB1/CUL4 E3 ubiquitin ligase (77, 82).

### HBx and Autophagy

Autophagy is an evolutionarily conserved process by which eukaryotic cells degrade disposable or potentially dangerous cytoplasmic material to maintain cellular homeostasis (97). Accumulating evidence has delineated that HBx can hijack the molecular machinery to modulate autophagy, contributing to enhanced viral replication, liver inflammation and carcinogenesis (98–101).

An earlier study revealed that HBx up-regulated beclin 1 expression to sensitize hepatic and hepatoma cells to starvation induced autophagy *in vitro*, which might provide an novel insight of hepatocytes growing under nutrient-deficient conditions in HBV infection (98). Moreover, in human hepatoma cells, HBx could bind to Vps34, the catalytic subunit of phosphatidylinositol 3-kinase class III (PI3KC3), thus activate PI3KC3, leading to autophagy which has a positive effect on HBV DNA replication (99).

Mitophagy is a specific kind of autophagy, it occupies a key position in the maintenance of mitochondria homeostasis. Mitophagy is a degradation process which selectively targets the impaired mitochondria to autophagosomes. BNIP3-like (BNIP3L) is a mitochondrial membrane protein which promotes the occurrence of mitophagy. Chen et al. have shown that HBx induces BNIP3L-dependent mitophagy *via* accelerating autophagic flux, by which glycolytic metabolism is up-regulated and cancer stemness of HCC cells is increased (100). Besides, tumor necrosis factor superfamily member 10 (TNFSF10) is a typical death ligand which binds to the death receptor TNFRSF10B/DR5 (TNF receptor superfamily member 10b), fostering immune surveillance against virus-infected cells. HBx could form a ternary complex with TNFRSF10B and LC3B (light chain 3B), subsequently inhibits TNFSF10 receptor signaling *via* autophagy-mediated degradation of TNFRSF10B, leading to virus evasion from TNFSF10-mediated immunity. This process mediated by HBx may potentiate HBV-induced liver inflammation and viral persistence (101).

### HBx and microRNA

**Figure 4:**
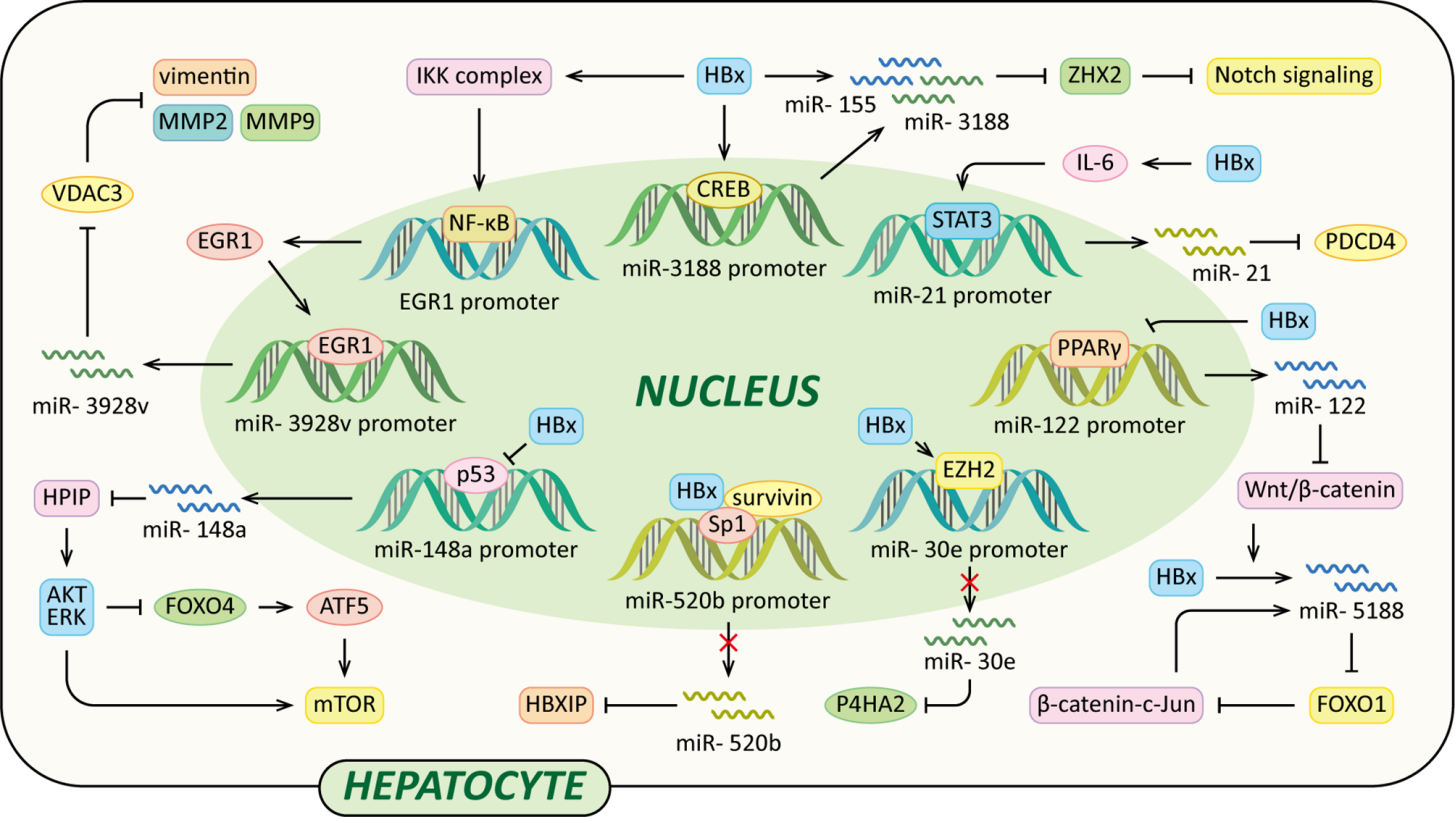
Regulatory models of HBx and miRNAs involved in liver pathogenesis. The mechanisms involved in HBx and miRNAs are related to Notch signaling pathway, Wnt/β-catenin signaling pathway, MMP, collagen deposition, HPIP/AKT/ERK/FOXO4/ATF5/mTOR signaling pathway and HBXIP, which result in cell proliferation, anti-apoptosis, c-Jun activation, VM, EMT, liver fibrosis and eventually HCC.

MicroRNAs (miRNAs) are a class of small (18-25nt) non-coding RNAs that mostly interact with the 3’ untranslated regions (UTRs) of their target mRNAs and modulate protein production of target genes at the post-transcriptional level (102). Over the past decade, many studies have revealed that dysregulation of various miRNAs have a role in the development of liver fibrosis and HCC, and part of which are induced by HBx (**Figure 4**).

It has been reported that miR-3188 expression levels are significantly up-regulated in HCC tissues, HBV transgenic mice, and HepG2.215 cells. And HBx promotes CREB-mediated activation of miR-3188 *in vitro* (103). Zinc-fingers and homeoboxes (ZHX) 2 acts as a transcriptional repressor of Notch1 *via* interaction with NF-YA (104). MiR-3188 binds with the 3’UTR of ZHX2 mRNA to down-regulate ZHX2, contributing to Notch signaling activation. Thus, the HBx–miR-3188–ZHX2-Notch1 signaling pathway might play a pivotal role in HBV-related HCC progression (103). HBx also represses ZHX2 protein levels by up-regulation of miR-155. The miRNA binds to seed sites in the 3’UTR of the ZHX2 mRNA and inhibits its translation (105).

A recent study also found that HBx induced miR-5188- forkhead box O1 (FOXO1)/β-catenin-c-Jun feedback loop by Wnt/β-catenin-c-Jun signaling. MiR-5188 could suppress the expression of E-cadherin and induce the expression of β-catenin, N-cadherin, vimentin and c-Jun, which could be reversed by FOXO1. MiR-5188 up-regulated by HBx could bind the sequences within the FOXO1 3’UTR to down-regulate FOXO1. Moreover, c-Jun could up-regulate miR-5188 transcription *via* binding to miR-5188 promoter region, thus forming a positive feedback loop to facilitate Wnt/β-catenin activation which could promote HCC stemness, metastasis, proliferation, and resistance to chemotherapy (106).

In addition, in HCC cells, HBx facilitates the activation of miR-3928v expression *via* increasing early growth response 1 (EGR1) expression and promoting its translocation into the nucleus in an NF-κB signaling-dependent manner. Voltage-dependent anion channels (VDAC3) is a member of mitochondrial porin family, it can suppress vascular mimicry (VM) and EMT by reducing MMP2, MMP9 and vimentin expression in HCC cells. MiR-3928v directly targets the 3’UTR of VDAC3 mRNA to down-regulate VDAC3. Collectively, the HBx-mediated up-regulation of miR-3928v results in the VDAC3 down-regulation which is a possible mechanism of HCC progression (107). It has been reported that HBx activates IL-6-STAT3 pathway to up-regulate miR-21 expression, which is critical for promoting hepatocarcinogenesis in HBV infection (108). Furthermore, programmed cell death 4 (PDCD4) exerts functions as a tumor suppressor, it could suppress tumor growth and induce apoptosis of human HCC cell lines to inhibit the development of HCC. MiR-21 up-regulated by HBx could suppress PDCD4 expression, contributing to HCC development (109).

Moreover, HBx increases methylation of CpG islands in the promoter of miR-30e in an EZH2-formed complexes-dependent manner to suppress the expression of miR-30e (110). Prolyl 4-hydroxylase subunit α2 (P4HA2) is an isoform of P4H α family which could facilitate overly crosslinked collagen generation to promote liver fibrosis development (110–112). MiR-30e could inhibit the P4HA2 expression *via* directly targeting its 3’UTR. The overexpression of P4HA2 mediated by down-regulation of miR-30e accelerates the collagen deposition in the liver *in vivo* and *in vitro*, ultimately leading to liver fibrosis and the growth of liver cancer (110). Additionally, a study performed by Xu et al. revealed that HBx suppressed miR-148a, which in turn activated the HPIP/AKT/ERK/FOXO4/ATF5/mTOR pathways to promote EMT, invasion and metastasis of HCC. P53 could bind to the promoter of miR-148a to activate miR-148a expression, while this process is suppressed by HBx *via* interacting with p53. MiR-148a binds to the 3’UTR of the HPIP (hematopoietic pre–B cell leukemia transcription factor–interacting) mRNA to down-regulate HPIP expression. HPIP interacts with p85 and Src to activate AKT and ERK which can inactivate FOXO4 by phosphorylation, subsequently up-regulating the expression of ATF5 (activating transcription factor 5) to activate mTOR (113). Another tumor suppressor miR-520b is down-regulated by HBx and its partner survivin *via* regulating transcription factor Sp1, leading to elevated expression of oncopreotein hepatitis B X-interacting protein (HBXIP) (114). Furthermore, It has been reported that miR-122 inhibits Wnt/β -catenin signaling pathway *via* directly suppressing WNT1 protein expression in HCC cells (115). HBx could inhibit miR-122 transcription in HCC cells by binding Peroxisome proliferator-activated receptor-gamma (PPARγ), which subsequently suppresses PPARγ-mediated transactivation of tumor suppressor miR-122 (116).

### HBx and lncRNA

**Figure 5:**
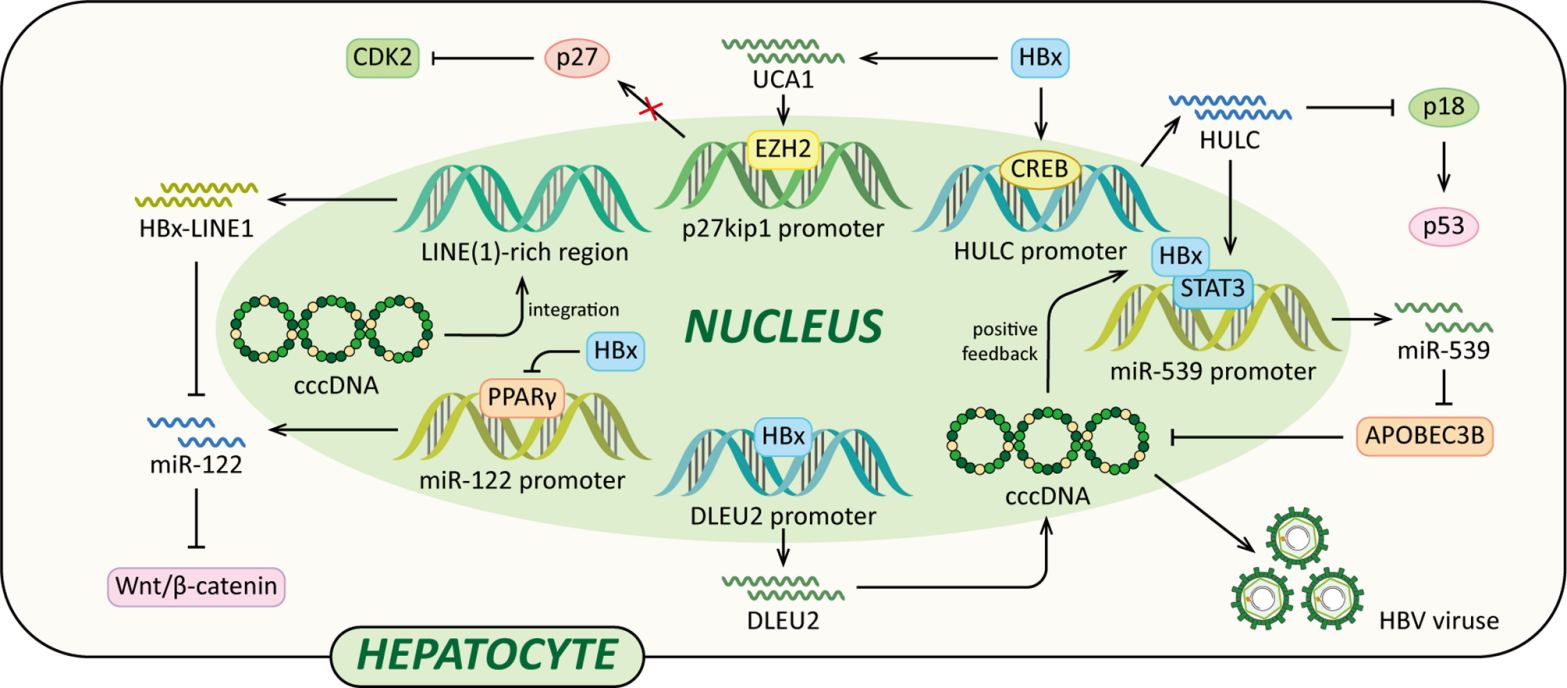
Regulatory models of HBx and lncRNAs involved in liver pathogenesis. The mechanisms involved in HBx and lncRNAs are related to epigenetic modulation, Vimentin, miR-122, Wnt/β-catenin signaling pathway, p18, miR-539 and p27, which cause elevated HBV transcription and replication, EMT, diminished p53, cccDNA stability, cell growth, anti-apoptosis and eventually HCC.

Long non-coding RNAs (lncRNAs) are a class of regulatory non-coding RNAs with larger than 200nt in length (117). Previously lncRNAs were disregarded as transcriptional “noise” (118). However, accumulating data have indicated that lncRNAs have many complicated functions, such as epigenetic modification, transcription, post-transcriptional modification and cellular signal transduction regulations. And in HBV-related HCC, lncRNAs are frequently dysregulated (119). Recently, it has been demonstrated that several lncRNAs, such as DLEU2, Dreh, highly up-regulated in liver cancer (HULC), HBx-LINE1 and UCA1, are involved in HCC development, and their aberrant expression is associated with HBx (**Figure 5**) (79, 120–125).

A recent study indicated that HBx could enhance lncRNA DLEU2 transcription by binding the promoter region of DLEU2 in HBV-infected hepatocytes (79). Histone trimethyltransferase EZH2 is the catalytic active subunit of the polycomb repressor complex 2 (PRC2) (79, 126). It catalyses the addition of H3K27me3 which is a repressive chromatin mark leading to the epigenetic silencing (126). Nuclear DLEU2 physically interacts with HBx and EZH2. The HBx-DLEU2 interaction and their recruitment on the cccDNA result in EZH2 displacement from the viral chromatin, hence boosting HBV transcription and replication. Furthermore, HBx and DLEU2 could coregulate a subset of host cancer-related genes to promote hepatocytes transformation (79).

In addition, a study using HBx transgenic mice demonstrated that HBx could down-regulate lncRNA Dreh to promote HCC metastasis (120). Dreh could serve as a tumor suppressor by targeting and altering type III intermediate filament Vimentin, the major cytoskeletal component of mesenchymal cells (120, 127). In mouse liver cells, Dreh could alter Vimentin to a relatively stable cytoskeleton structure to reverse the high-migration phenotype of HCC cells, therefore suppressing cell invasion and migration. Taken together, the down-regulation of Dreh mediated by HBx provides a possible rationale for the development of HBx-related HCC (120).

Moreover, HBx activates lncRNA HULC promoter *via* CREB to up-regulate HULC, which promotes hepatoma cell proliferation *via* down-regulating tumor suppressor p18 (121). The expression of p18 is usually down-regulated in HCC, p18 can translocate to the nucleus to activate p53 through the interaction with ataxia telangiectasia-mutated (ATM) in response to DNA damage (128). In addition, HULC could significantly up-regulate miRNA-539 by coactivating the transcription factor STAT3 with HBx. MiRNA-539 targets the 3’UTR of DNA cytidine deaminase APOBEC3B mRNA to down-regulate the APOBEC3B in hepatoma cells (122). Thus, the suppression of APOBEC3B enhances HBV cccDNA stability and promotes HBV replication, leading to up-regulation of HBx. Therefore, a positive feedback loop of HULC/HBx/STAT3/miR-539/APOBEC3B is formed, which might contribute to the growth of liver cancer.

Lau et al. reported that HBx-LINE1, a specific chimeric transcript, exerted functional effects as a lncRNA. HBx-LINE1 activates Wnt/β-catenin signaling and promotes cell motility through EMT in human HBV-infected hepatoma cells (123). Furthermore, HBx-LINE1 acts as a molecular sponge absorbing cellular miR-122 in Huh7 cells, each HBx-LINE1 contains six miR122-binding sites, facilitating hepatic cell EMT-like changes *via* depleting cellular miR-122. And it promotes mouse liver inflammation and injury (124). Furthermore, it is reported that the signaling pathway of the HBx-UCA1/EZH2-p27Kip1 axis is a potential mechanism of hepatocarcinogenesis. Protein p27 is a cyclin-dependent kinase (CDK) 2 inhibitor. In LO2 and Hep3B cells, HBx up-regulates lncRNA UCA1 which epigenetically silences p27 expression by physical association with EZH2, leading to promoted cell growth and inhibited apoptosis (125).

## Conclusion and Perspectives

The mechanisms of HBV infection chronicity are still not well understood, but they might be multifactorial and related to inadequate immune response. The HBV encoding proteins may play a crucial part in the chronicity of HBV infection, for example, they take advantages of host immune system and use many other strategies to facilitate the long-time persistence of viruses. The recent studies have generated significant amount of information for understanding how the HBV encoding proteins take advantage of the host immune system to facilitate HBV persistence and promote liver pathogenesis *via* various strategies, such as transcriptional activation, epigenetic modifications, ubiquitination, autophagy and non-coding RNA (ncRNA) regulation.

However, a few functions of these proteins seem like disadvantages for the viral persistence, such as HBcAg mediates the degradation of cccDNA by interacting with APOBEC3A and HBV DNA polymerase attenuates HBV replication *via* CREB1-HOTTIP-HOXA13 axis, resulting in repressed viral replication (43, 63). These functions might be considered as negative feedback pathways of HBV replication by which HBV virus attenuates host cell injury to promote the chronicity of infection, but they also could be viewed as a unique possibility for new treatments to abolish viral persistence.

Of note, the current treatments for CHB patients, such as NUCs and IFN-α or its pegylated derivative, are less than satisfactory. Considered CHB as a result of HBV induced immune tolerance, there is a need for developing immunotherapy strategies to break immune tolerance and establish long-term control of infection. Activating innate immune receptors such as TLRs and retinoic acid-inducible gene 1 (RIG-I) is one of the developing therapeutic approaches for CHB. TLR agonists have been developed in recent years on the base of positive results from preclinical studies, for example such as, TLR7 agonist RO6864018 and RO7020531 by Roche and TLR8 agonist GS-9688 by Gilead. They are orally available, small-molecule agonists under clinical trials and expected to activate TLRs on immune cells in the liver. Antiviral responses against HBV can be induced by RIG-I activation. Currently, RIG-I agonists SB 9200 and GS-9992 are being tested in phase 2 clinical trials. Moreover, the key to control HBV infection appears to activating HBV-specific immune responses. In the past years, strategies targeted on adaptive immune responses have been explored, such as checkpoint inhibitors that boost antigen-specific T cells, genetically edited T cells (chimeric antigen receptor T (CAR-T) and T cell receptor (TCR)-T cells) and diverse therapeutic vaccines (129, 130). The improvement of our knowledge on the functions of these viral encoding proteins could provide the rationale to interfere the process of HBV pathogenesis with the goal of control HBV infection.

In addition, compared with adults, HBV infection in children is featured by an immunological tolerance which is chronicity-prone. Therefore, further research on HBV infection in children is required to understand immune evasion mechanisms associated with HBV encoding proteins also seen in adults. Contrary to adults, the immune system of infants and children is unable to induce an adequate HBV specific immune response (131). About 90% of HBV infection in neonates and infants would develop into chronic infection, while less than 5% in adults who acquire infection. Most adults with CHB were infected *via* mother-to-child transmission perinatally or during early childhood (132). Although many studies on HBV induced immune dysfunction have published in recent years, the underlying mechanisms of CHB resulting immunotolerance in infancy and childhood are still poorly understood. Therefore, additional research of immunopathogenesis in children with HBV is needed, better understanding of how HBV encoding proteins exert functions in children could help us prevent HBV chronicity, establish treatment strategies and develop other new curative therapies.

However, our knowledge of HBV encoding proteins is still hindered by technical limitations. There are difficulties in obtaining data of patients, especially natural infection patients who are at the presymptomatic stages. Many of HBV related studies were conducted in *in vitro* models, most of them use cell culture systems which are convenient. These experimental results are basically based on expression of one HBV encoding protein, and most mechanisms have not been confirmed in HBV patients. Meanwhile, even *in vitro* experiments are also hampered by the limited availability of non-transformed hepatocytes *in vitro* HBV infection study system, these non-transformed hepatocytes have a limited life span and their infection efficiency and HBV replication level are low (133–135). Despite HBV infection animal models could provide certainly useful data of physiology, they are also limited by ethical issues and sometimes high costs (chimpanzees), as well as difficulties to obtain mouse models which are fully permissive to HBV infection (135). Therefore, new and improved models for the development of HBV studies are in urgent need.

## Author Contributions

Conception and design: JQ and QZ. Collection and assembly of data: FZ, XX, MT, and HL. Data analysis and interpretation: FZ and HY. Manuscript writing: FZ, JQ, and QZ. Manuscript revision: FZ, XT and JQ. Administrative support: QZ and CQ. All authors contributed to the article and approved the submitted version.

## Funding

This work was supported in part by grants from the National Natural Science Foundation of China (81770607, 81772626, 81600469, 81570551), the Major Special Plan of Science and Technology of Shandong Province (2015ZDXX0802A01), the Clinical Medical Science and Technology Innovation Program (202019094), and WBE Liver Fibrosis Foundation (CFHPC2021011).

## Conflict of Interest

The authors declare that the research was conducted in the absence of any commercial or financial relationships that could be construed as a potential conflict of interest.

## Publisher’s Note

All claims expressed in this article are solely those of the authors and do not necessarily represent those of their affiliated organizations, or those of the publisher, the editors and the reviewers. Any product that may be evaluated in this article, or claim that may be made by its manufacturer, is not guaranteed or endorsed by the publisher.
